# Novel factors of *Anopheles gambiae* haemocyte immune response to *Plasmodium berghei* infection

**DOI:** 10.1186/s13071-016-1359-y

**Published:** 2016-02-09

**Authors:** Fabrizio Lombardo, George K. Christophides

**Affiliations:** Department of Life Sciences, Imperial College London, London, UK; Current address: Department of Public Health and Infectious Diseases, Sapienza University of Rome, Rome, Italy

**Keywords:** *Anopheles gambiae*, Innate immunity, *Plasmodium*, RNAi, Melanisation

## Abstract

**Background:**

Insect haemocytes mediate cellular immune responses (e.g., phagocytosis) and contribute to the synthesis of humoral immune factors. In previous work, a genome-wide molecular characterization of *Anopheles gambiae* circulating haemocytes was followed by functional gene characterization using cell-based RNAi screens. Assays were carried out to investigate the role of selected haemocyte-specific or enriched genes in phagocytosis of bacterial bio-particles, expression of the antimicrobial peptide cecropin1, and basal and induced expression of the mosquito complement factor LRIM1 (leucine-rich repeat immune gene I).

**Findings:**

Here we studied the impact of a subset of genes (37 candidates) from the haemocyte-specific dsRNA collection on the development of *Plasmodium* in the mosquito by in vivo gene silencing. Our screening identifies 10 novel factors with a role in the mosquito response to *Plasmodium*. Analysis of in vivo screening phenotypes reveals a significant anti-correlation between the prevalence of oocysts and melanised ookinetes.

**Conclusions:**

Among novel immune genes are putative pattern recognition proteins (leucine-rich repeat, fibrinogen-domain and R-type lectins), immune modulation and signalling proteins (LPS-induced tumor necrosis factor alpha factor, LITAF and CLIP proteases), and components of extracellular matrix such as laminin and collagen. Additional identified proteins such as the storage protein hexamerin and vesicular-type ATPase (V-ATPase) are associated for the first time with the mosquito response against *Plasmodium*.

**Electronic supplementary material:**

The online version of this article (doi:10.1186/s13071-016-1359-y) contains supplementary material, which is available to authorized users.

## Findings

### Background

*Plasmodium* parasites must overcome several barriers before they can successfully establish infection in their anopheline mosquito vector. They include the microbiological barrier of the mosquito midgut microbiota, two physical barriers involving the peritrophic matrix and the midgut epithelium, and the immunological barrier of the mosquito innate immune system. The latter plays a critical role immediately after a *Plasmodium* ookinete crosses the midgut epithelium and before it develops into an oocyst. Circulating haemocytes are important contributors to the haemolymph immune response [1, 2]. They take part in defense against invading microorganisms, both through cellular processes like phagocytosis and through the production and secretion of soluble humoral factors, such as antimicrobial peptides, complement-like proteins and components of proteolytic enzymes that control melanisation [3, 4].

To identify novel factors of the mosquito immune response and derive further insights into the function of haemocytes, we have recently developed in vitro, cell-based, double-stranded RNA (dsRNA) screens of about 100 *Anopheles gambiae* genes specifically or predominantly expressed in haemocytes [5]. Using these screens, we have identified several novel modulators of phagocytosis, antimicrobial peptide expression, and expression of the complement factor, LRIM1. Here, we use a subset of this dsRNA collection to identify genes affecting *An. gambiae* infection with the rodent parasite *Plasmodium berghei* in vivo. This study extends our earlier published work and concludes the in vivo screening [2]. The data obtained from our screen are integrated with in vitro results obtained previously, highlighting a role of several genes in haemocyte innate immune responses to *Plasmodium* infection.

### In vivo *RNAi screen to identify* Plasmodium *modulators*

We selected a subset of 39 dsRNAs corresponding to 37 putative immune modulators from an *An. gambiae* haemocyte-specific dsRNA library we had previously generated (Additional file 1: Table S1). These genes exhibit enriched expression in haemocytes, are differentially regulated by immune challenges, and/or have immune-related InterPro domains, signal peptides or transmembrane domains [2, 5]. Experimental procedures, such as gene knock down (KD), mosquito infections with *P. berghei* and parasites assessment in the midguts, were performed according to standard protocols, detailed in Additional file 2 (primers used are listed in Additional file 3: Table S2). KD efficiency was assessed for a representative group of candidates and results are summarized in Additional file 4: Table S3.

Four successive screening rounds were implemented of 39, 29, 20 and 3 dsRNAs, respectively. DsRNAs were included in the next round of testing if they showed significant effects or at least a constant trend on either parasite intensity or prevalence of live oocysts or melanised ookinetes. Quality control and replicate pooling criteria were applied before performing statistical analyses (described in Additional file 2). Results are summarized in Table 1 and records of parasite counts of each gene KD are reported in Additional file 5: Table S4.

**Table 1:** In vivo RNAi screen results. Gene KDs affecting the number of developing oocysts and the prevalence of developing oocysts and/or melanised ookinetes are listed. Descriptive statistics (arithmetic mean ± standard error) and P values as results of statistical tests to compare parasite intensity and prevalence of each group with corresponding LacZ control are reported here. Upper part of the Table (above the double line): genes affecting oocyst intensity; lower part of the Table (below the double line): genes affecting prevalence of infection and/or oocyst / melanised ookinete intensities. Significant P values (*P* < 0.05) are reported. ns: *P* > 0.05

			Developing oocysts					Melanised ookinetes				
			*Intensity*			*Prevalence*		*Intensity*			*Prevalence*	
Gene KD	Rep.	N.	Mean ± SE	Fd	*P*§	%	*P*¶	Mean ± SE	Fd	P§	%	*P*¶
AGAP010658°	3	51	64.4 ± 11.8	3.1	0.0004	86	ns	0.2 ± 0.2	0.1	0.035	5	0.048
LacZ		63	20.8 ± 4.4			80		2.4 ± 1.0			18	
AGAP007540	3	45	77.3 ± 12.1	2.1	0.002	84	ns	1.4 ± 0.6	0.6	ns	13	0.008
LacZ		64	37.3 ± 6.2			83		2.2 ± 1.1			29	
AGAP009201	3	58	56.6 ± 9.4	1.9	0.032	81	ns	9.0 ± 5.4	6.5	0.040	25	ns
LacZ		84	30.5 ± 5.0			87		1.4 ± 0.6			17	
AGAP004017	3	62	23.7 ± 5.0	0.7	0.036	69	ns	5.1 ± 1.7	0.6	ns	39	ns
LacZ		59	34.8 ± 5.5			76		8.0 ± 5.2			27	
AGAP004928	3	64	32.5 ± 4.9	0.7	0.042	81	ns	3.5 ± 1.7	3.9	ns	24	ns
LacZ		68	45.9 ± 5.8			95		0.9 ± 0.3			19	
AGAP004993	4	60	32.7 ± 7.9	0.6	0.002	69	0.0001	11.3 ± 4.4	1.5	ns	33	ns
LacZ		58	54.0 ± 7.5			85		7.3 ± 2.7			39	
AGAP003960	3	52	18.7 ± 3.8	0.5	0.044	71	ns	4.0 ± 0.8	0.2	ns	48	ns
LacZ		47	38.5 ± 7.6			78		16.5 ± 7.1			39	
AGAP011223	3	51	60.7 ± 12.9	1.4	ns	80	0.0002	9.6 ± 5.3	3.2	ns	22	0.018
LacZ		47	44.8 ± 11.5			96		3.0 ± 1.1			36	
AGAP003879	3	76	40.2 ± 8.3	0.9	0.011	69	0.026	8.6 ± 2.6	1.5	0.003	40	0.0018
LacZ		71	44.3 ± 5.6			87		5.9 ± 4.3			19	
AGAP012034	3	37	70.7 ± 25.9	2.0	ns	76	ns	0.7 ± 0.7	0.3	0.005	4	0.0001
LacZ		47	35.9 ± 9.5			81		2.6 ± 0.9			29	

Gene KD: ID of silenced genes (VectorBase Gene IDs)

Rep.: number of independent replicates

N.: sample size (total number of mosquitoes across replicates)

Mean ± SE: arithmetic mean ± standard error (SE) of parasite intensities per midgut in each group after pooling replicate data

Fd: fold difference, ratio between mean oocyst (or melanised ookinete) value of a specific gene KD and the LacZ KD

P§: statistical significance according to Mann–Whitney *U*-Test on oocyst or melanised ookinete intensity of pools (gene-specific KD vs LacZ KD)

%: prevalence (infected mosquitoes/total mosquitoes) of developing oocysts or melanised ookinetes calculated as geometric means of the prevalence of each replicate

P¶: statistical significance according to Fisher’s Exact Test on oocyst or melanised ookinete prevalence (gene-specific KD vs LacZ KD)

° SNAP_ANOPHELES00000017730

Silencing *AGAP007540*, *AGAP009201* and *SNAP_ANOPHELES00000017730* (long version of VectorBase predicted *AGAP010658*, henceforth AGAP010658*) resulted in a significant increase of oocyst intensities (and also melanised ookinete intensities, as for *AGAP009201*). *AGAP007540* and AGAP010658* silencing also led to a significant decrease in melanised ookinete prevalence (and intensity as for AGAP010658*). Silencing *AGAP003960*, *AGAP004017*, *AGAP004928* and *AGAP004993* caused a decrease in oocyst intensity. *AGAP004993* silencing also significantly decreased the oocyst prevalence. *AGAP011223* silencing decreased oocyst and melanised ookinete prevalence, while *AGAP003879* silencing resulted in a decrease of oocyst intensity and prevalence and an increase of melanised ookinete intensity and prevalence. Lastly, silencing *AGAP012034* significantly reduced the intensity and prevalence of melanised ookinetes and increased the number of developing oocysts.

### *Novel modulators of the mosquito immune response to* Plasmodium

The RNAi screen of 37 genes specifically or predominantly expressed in *An. gambiae* haemocytes identified ten novel modulators of mosquito infection with *P. berghei*. Below is a brief summary of the main characteristics of these genes, such as sequence similarities with known immune factors or domains and comparisons with phenotypes of orthologs in other insects (see Additional file 1).

*AGAP007540* encodes a putative von Willebrand factor-type A domain (vWF) protein. The vWF domain can serve various biological functions in insects including haemolymph coagulation and haemostasis, wound healing and other innate immunity functions [6].

*AGAP009201* is highly expressed in circulating haemocytes [2] and encodes for a collagen type IV protein, thought to be involved in the extracellular matrix, such as the basal lamina. Laminin and collagen are components of the basal lamina and interact with invading parasites [7]. Previous in vivo and cell-based RNAi assays have shown that laminin silencing leads to reduced oocyst intensity and increased phagocytosis capacity [5]. A role of laminin was proposed in regulating the expression of the complement factor *LRIM1* during an immune challenge [5]. Here we reveal that additional putative components of the basal lamina are involved in these reactions, as recently described in the greater wax moth, *Galleria mellonella* [8], and the flour beetle *Tribolium castaneum* [9].

AGAP010658* encodes a homolog of the hexamerin 2 beta of *An. darlingi* Root and *Aedes aegypti* (Linneaus) and the larval serum protein 1 (LSP1.1) of *Culex quinquefasciatus* Say, which serve as major storage proteins [10]. The strong activation after blood meal suggests that hexamerins are a source of amino acids for the synthesis of vitellogenin in the fat body. A function of storage proteins and vitellogenin (Vg) in various facets of arthropod innate immunity has been described [11]. It has also been shown that depletion of the lipid carrier protein lipophorin (Lp) reduces the number of developing *Plasmodium* oocysts in the mosquito midgut, while both *An. gambiae* Lp and Vg are required for the function of the complement factor TEP1 (thioester-containing protein 1) against *Plasmodium* ookinetes [12].

*AGAP003960* encodes a putative transmembrane protein encompassing peptidase and trypsin-like domains, possibly involved in immune regulation through proteolytic processing. *AGAP003960* transcripts are enriched in haemocytes and up-regulated upon bacterial challenge in mosquito cell cultures [13].

The Leucine-Rich Repeat (LRR) domain protein-encoding gene, *AGAP004017*, is specifically expressed in circulating haemocytes [2]. It carries a predicted signal peptide and a transmembrane domain, and does not belong to the LRIM family of proteins [14]. *AGAP004017* clusters with *AGAP004016*, another LRR-containing protein that is also highly expressed in haemocytes and acts as a *Plasmodium* agonist [2].

*AGAP004928* (*LL6*) encodes a LITAF (LPS-induced tumor necrosis factor alpha factor) domain, a membrane-associated motif possibly involved in immune signalling pathways [15]. We previously showed that this gene plays a role in bacterial phagocytosis [5]. Recently, additional members of this family were associated with the defence against *Plasmodium* [16]. Indeed, *An. gambiae* LITAF-like 3 (*LL3* - *AGAP009053*) expression is up-regulated in response to midgut invasion by both rodent and human malaria parasites, and its KD analysis reveals a role in anti-*Plasmodium* defence [17]. Four members of the LITAF family i.e. *LL1, LL2, LL3* and *LL4* are closely related, while *LL5* and *LL6* are more divergent. *LL6* (*AGAP004928*) clusters with *Drosophila melanogaster CG13559*, a member of the fruit fly LITAF family expressed in the haemocytes and modulated by immune challenge [18].

*AGAP004993* encodes an *An. gambiae* homolog of *D. melanogaster laminin* (*LanA*), an extracellular matrix protein with several functions. Six laminin paralogs are found in the *An. gambiae* genome: *netrin 1* (*AGAP000228*), *netrin 1* homolog (*AGAP000225*), *laminin gamma 1* (*AGAP007629*), *multiple epidermal growth factor-like domains 10* (*AGAP007256*), *laminin alpha 1/2* (*AGAP007849*) and *laminin beta 1* (*AGAP001381*). We previously showed that the latter regulates both phagocytosis and basal and induced expression of *LRIM1* [5], while *laminin gamma 1* and *LanB2* (*AGAP007629*, Q9U3U7) were shown to promote oocyst development in the mosquito midgut, possibly by inhibiting their melanotic encapsulation [19].

*AGAP011223* encodes the fibrinogen-related *FBN8* (also known as *FREP57*) that is shown to play a role in anti-*Plasmodium* defence [20]. We previously demonstrated that *FBN8* promotes phagocytosis of bacterial bio-particles [5], highlighting the complex networks regulating mosquito innate immunity.

*AGAP003879* encodes a vesicular-type ATPase, a transmembrane protein involved in several cellular processes [21]. V-ATPase utilizes ATP to actively transport H^+^, regulating osmotic changes in mosquito cells and osmoregulatory tissues, including the stomach, malpighian tubules, gut and rectum. A role of a V-ATPase in *Plasmodium* infection in *Aedes* and *Anopheles* was previously suggested, since the typical distribution of oocysts in the posterior half of the midgut overlaps with the spatial distribution of V-ATPase-overexpressing epithelial cells [22]. We previously showed that KD of V-ATPase reduces phagocytosis of *E. coli* bio-particles [5]. Here we reveal for the first time that KD of V-ATPase decreases the oocyst intensity and prevalence. It remains to be elucidated whether this effect of V-ATPase is caused by altered cellular equilibrium of water and ions (as for phagosome acidification and maturation) or by more conventional immune mechanisms.

Finally, *AGAP012034* encodes a potential new member of the subfamily B of CLIP-domain serine proteases. The role of CLIPBs and their putatively inactive homologs, CLIPAs, as activators or suppressors of the *An. gambiae* melanisation response against *P. berghei* is well known [23]. *AGAP012034* maps to a genomic cluster of four highly conserved CLIPBs, including *CLIPB20* that is regulated after *Serratia marcescens* infection [24].

In conclusion, our results identify 10 novel regulators of the haemocyte immune response to *Plasmodium*. A complex role in different immune responses is discovered for some proteins (for instance, V-ATPase and LL6), as it arises by comparing results of this work with KD phenotypes from previous screens [5]. Finally, additional proteins such as the storage protein hexamerin and the V-ATPase are associated for the first time with the mosquito response against the malaria parasite.

### Parasite melanisation is linked to parasite killing

**Fig. 1:**
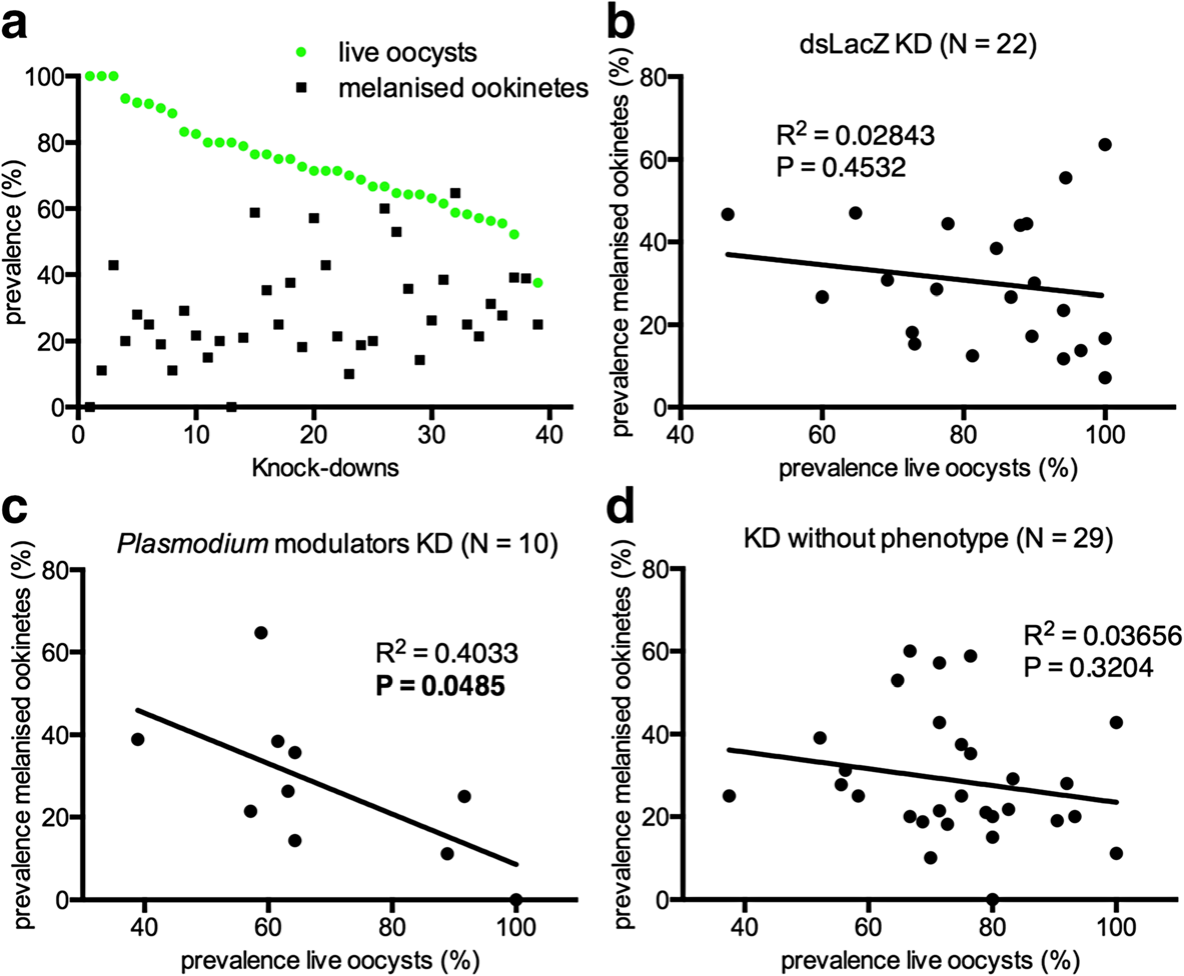
Correlation between prevalence of live oocysts and melanised ookinetes. A. Oocyst prevalence data from the first round of screening of 39 dsRNA targeting 37 genes sorted from highest (100 %) to lowest (37.50 %). Linear regression and correlation between prevalence of live oocysts and melanised ookinetes assessed in: B, 22 groups of mosquitoes injected with dsLacZ, which were used as controls in the four rounds of screenings (correlation coefficients: Pearson r: −0.1686; Spearman r: −0.2376, *P* = 0.2869); C, 10 groups of mosquitoes injected with dsRNAs that showed effects on parasite development (correlation coefficients: Pearson r: −0.6351; Spearman r: −0.6991, *P* = 0.027) and D, 29 groups of mosquitoes injected with dsRNAs that did not show effects on parasite development (correlation coefficients: Pearson r: −0.1912; Spearman r: −0.2727, *P* = 0.1523). Insets in graphs B, C and D report Pearson R^2^ values and the relative statistical significance (P)

We assessed the overall correlation of the prevalence of oocysts and melanised ookinetes across the dataset. By sorting one of the two phenotypes from highest to lowest, we observed a clear trend of anti-correlation between the two variables (Fig. 1a) that led us to partition the data according to whether effects on parasite development were observed or not. Data obtained in the first round of screening for 39 dsRNA targeting 37 genes were considered (Fig. 1a, c and d) as well as data from 22 dsLacZ injections in the four successive rounds of screenings to be used as a reference (Fig. 1b). A statistically significant anti-correlation between the two phenotypes was detected when genes that produced KD phenotypes were analysed (Fig. 1c) while no correlation was observed when genes that did not cause KD phenotypes and dsLacZ controls were evaluated (Fig. 1b and d). The dual role of melanisation in both parasite killing and clearance has been well established [25–27]. Our data corroborate the function of melanisation as a clearance mechanism that follows parasite killing by the mosquito immune system. Recently, novel insights into the balance between immune tolerance and resistance, as well as into the ability of the innate immune system to recognize and combat different pathogens using different strategies allowed the definition of innate immunity as a combination of both microbe clearance (melanisation) and damage control (pathogen survival) [28].

### Ethics statement

Animals were cared for in accordance with the guidelines reported in the revised Animals (Scientific Procedures) Act 1986 (UK).

## Additional files

Additional file 1: Table S1. dsRNA haemocyte-specific library. The main features of target genes and dsRNAs used in this study are presented. DsRNA IDs, KD phenotypes in cell-based RNAi screens [5], VectorBase gene IDs, Affymetrix probe codes and previous ENSEMBL IDs are listed in columns A, B, C, D and E, respectively. Circulating haemocyte microarray information from [2] are summarized in columns F (cluster), G (normalized haemocyte value), H (normalized carcass value) and I (normalized head value). Comments (name and/or homology of *An. gambiae* genes) and InterPro domain data are reported in columns J and K, respectively. *Drosophila melanogaster* orthologs are shown in column L, and corresponding FlyBase IDs in column M. *D. melanogaster* KD phenotypes according to the GenomeRNAi database are reported in column N. This table is modified from Lombardo et al. 2013 [5]. (XLS 81 kb) Additional file 2:
**Detailed description of the experimental procedures and statistical analyses utilized.** Evaluation of gene knock down efficiency. (PDF 62 kb) Additional file 3: Table S2. List of primers used in this study. (PDF 86 kb) Additional file 4: Table S3. Measurement of knock down efficiency. (PDF 53 kb) Additional file 5: Table S4. Oocysts and melanised ookinetes dataset. (XLSX 195 kb)
