# Supplementary material for: Novel factors of Anopheles gambiae haemocyte immune response to Plasmodium berghei infection
Source: Parasit Vectors. 2016 Feb 9;9:78. doi: 10.1186/s13071-016-1359-y (PMC4746906; doi:10.1186/s13071-016-1359-y)
Supplement: Additional file 2: — Detailed description of the experimental procedures and statistical analyses utilized. Evaluation of gene knock down efficiency. (PDF 62 kb) [file 13071_2016_1359_MOESM2_ESM.pdf]

Detailed materials and methods

Mosquito rearing, dsRNA injections and Plasmodium berghei *infections*

Anopheles gambiae G3 strain was maintained according to standard insectary procedures (www.mr4.org/). DsRNA injections in 2-day-old mosquitoes were performed as described previously [Blandin S. *et al*., 2002. Reverse genetics in the mosquito *An. gambiae*: targeted disruption of the Defensin gene. EMBO reports vol. 3, no. 9, pp 852–856]. Four days post injection (dpi), mosquitoes were allowed to feed on mice infected with *P. berghei* GFP-CON 259cl2 strain [described in: Franke-Fayard B, *et al*., 2004. A *Plasmodium berghei* reference line that constitutively expresses GFP at a high level throughout the complete life cycle. Mol Biochem Parasitol 137(1):23-33]. Parasitemia was determined and mice with a parasitemia between 8-12% were used for mosquito infectious feeding. Oocyst prevalence and load were determined in dissected midguts (8 –10 dpi) by fluorescence microscopy.

DsRNA synthesis, RNAi procedures and quantitative RT-PCR

T7 primers were designed using the E-RNAi webservice at http://www.dkfz.de/signaling/e-rnai3/evaluation.php. PCR products were synthesized by amplification of cDNA from total females (see below) and purified using the QIAquick PCR Purification kit (QIAgen); dsRNA synthesis was performed according to MEGAscript T7 Kit (Ambion) protocol and the purified dsRNAs were purified by phenol/chloroform extraction; the quantity and the quality of dsRNAs were checked by agarose gel electrophoresis and Nanodrop readings, respectively.

Quantitative RT-PCR was performed using an Applied Biosystems ABI 7700 RealTime PCR machine, and the SYBR Green PCR Master Mix from Applied Biosystems according to standard procedures and manufacturers instructions. Expression levels were calculated by the relative standard curve method using S7 as endogenous control. Total RNA from KD females was extracted using Trizol Reagent (Invitrogen). After DNAseI (Invitrogen) treatment, first strand cDNA was synthesized using polyT primers (Invitrogen) and MMLV Reverse Transcriptase (Invitrogen).

Primers used are listed in Additional file 3.

Statistical analysis

Prism software (version 6.00, GraphPad Software) was used for statistical analyses. Each experiment was repeated at least three times. Quality control (QC) filters: only experiments with at

least 15 surviving mosquitoes per dsRNA (and dsLacZ) treatment (i.e., dsRNA injection followed by effective infected blood meal, with at least 10 dissected guts/dsRNA suitable for parasite counting) were considered as valid replicates; a parasite (live oocysts and melanised ookinetes) prevalence of at least 75% for both dsLacZ-treated and dsRNA-treated mosquitoes was set as the threshold to select replicates suitable for the establishment of pools for further statistical analysis.

As mentioned in the text, four successive screening rounds were implemented of 39, 29, 20 and 3 dsRNAs, respectively. DsRNAs were included in the next round if they showed statistically significant phenotypes (live oocyst/melanised ookinete intensities of dsRNA compared to dsLacZ according to non-parametric Mann-Whitney U-Test) or at least a constant trend on either parasite load or prevalence of live oocysts or melanised ookinetes. Pooling criteria: experiments were tested for statistical differences within treatments across replicates (as described in: [Riehle M.M. *et.al.*, 2008. *Anopheles gambiae* APL1 Is a Family of Variable LRR Proteins Required for Rel1- Mediated Protection from the Malaria Parasite, *Plasmodium berghei*. PLoS ONE 3(11): e3672. doi:10.1371/journal.pone.0003672]), applying non-parametric Kruskal Wallis ANOVA on Ranks or non-parametric Mann-Whitney and Kolmogorov-Smirnov tests: groups were established only when replicates matched QC criteria described above and statistical tests within treatments resulted negative ($P>0.01$). Normality tests (D'Agostino-Pearson and Shapiro-Wilk) were performed on both single replicates and pools, with most of the datasets showing non-parametric distribution. After pools were generated, descriptive statistical information was extracted, i.e., geometric and arithmetic means of oocyst and melanised ookinete intensities and prevalence rates. To compare dsLacZ with dsRNA phenotypes the non-parametric Mann-Whitney U-Test on oocyst or melanised ookinete intensities was applied. Geometric means were used to compare oocyst and melanised ookinete prevalence rates, and Fisher's exact test was applied to evaluate statistical significance.

Pearson and Spearman correlation coefficients were calculated to investigate the correlation between live oocyst and melanised ookinete prevalence data in different groups of KDs. Coefficient of determination (R^2) and the statistical probability (P) that correlations observed occurred not by chance were also evaluated.

Efficiency of gene knock down

The efficiency of gene specific silencing was assessed by qRT-PCR in a randomly chosen group of genes. dsRNAs targeting *AGAP004928*, *AGAP005227*, *AGAP009201*, *SNAP_ ANOPHELES00000017730*, *AGAP010658* and against LacZ were injected into 2-day old female mosquitoes and the expression of silenced genes was measured by qRT-PCR 4 days after dsRNA treatments. Three replicates for each gene KD were performed. Data were normalized to *S7* levels and calibrated to the gene-specific expression in dsLacZ treated mosquitoes. Results of KD efficiency of AGAP004928, AGAP005227 and AGAP009201 are reported in Lombardo *et al.*, 2013 [Lombardo F, Ghani Y, Kafatos FC, & Christophides GK (2013) Comprehensive Genetic Dissection of the Hemocyte Immune Response in the Malaria Mosquito *Anopheles gambiae*. PLoS Pathog 9(1):e1003145].
