# Supplementary material for: Novel factors of Anopheles gambiae haemocyte immune response to Plasmodium berghei infection
Source: Parasit Vectors. 2016 Feb 9;9:78. doi: 10.1186/s13071-016-1359-y (PMC4746906; doi:10.1186/s13071-016-1359-y)
Supplement: Additional file 3: Table S2. — List of primers used in this study. (PDF 86 kb) [file 13071_2016_1359_MOESM3_ESM.pdf]

Additional Table S2. List of primers used in this study.

dsRNA	AGAP	Primer Forward	Sequence	Primer Reverse	Sequence
A1	AGAP000182	AGAP000182_F	TAATACGACTCACTATAGGGCAGCATATATGTGCGGGTTG	AGAP000182_R	TAATACGACTCACTATAGGGCGACGAAAAGATGGTTGAT
A2	AGAP000220	AGAP000220_F	TAATACGACTCACTATAGGGCAGCGGTACAACTTCAACA	AGAP000220_R	TAATACGACTCACTATAGGGCAGGCTGGAGTTTTTGTTCC
A3	AGAP000249	AGAP000249_F	TAATACGACTCACTATAGGGATCTGATCGTGTGCTACGC	AGAP000249_R	TAATACGACTCACTATAGGGCTCTTTGTGCAGCTTGATG
A4	AGAP000305	AGAP000305_F	TAATACGACTCACTATAGGGAGCTGGGTGAGAGATGTTGG	AGAP000305_R	TAATACGACTCACTATAGGGTCAGATCGTCCTCCTCCAG
A5	AGAP000385	AGAP000385_F	TAATACGACTCACTATAGGGACATCTGTTCTTCCGCGTCT	AGAP000385_R	TAATACGACTCACTATAGGGTCCTGCTCGGAGAGAGAGAG
A6	AGAP001954	AGAP001954_F	TAATACGACTCACTATAGGGACAAGCTGCACGATAAT	AGAP001954_R	TAATACGACTCACTATAGGGTCCACAAGTCCAGCACAAAG
A7	AGAP002243	AGAP002243_F	TAATACGACTCACTATAGGGCGAACATGCAAAACAGCAAG	AGAP002243_R	TAATACGACTCACTATAGGGTGCAGTGATCGGTCAGAAAG
A8	AGAP002415	AGAP002415_F	TAATACGACTCACTATAGGGTGTCGTTATGCTCGAACAGC	AGAP002415_R	TAATACGACTCACTATAGGGCAGCGTTTCGTTGAAGATGA
A9	TCLAG163691	AGAP003878_F	TAATACGACTCACTATAGGGAAGCTCTATCTGGCCCACAA	AGAP003878_R	TAATACGACTCACTATAGGGCAGAACATCTCTGGGTGGT
A10	AGAP003879	AGAP003879_F	TAATACGACTCACTATAGGGCCTTGGTTGAGGCACAGATT	AGAP003879_R	TAATACGACTCACTATAGGGTACGAGCCGATGATGATGAA
A11	AGAP003898	AGAP003898_F	TAATACGACTCACTATAGGGTTCCTGCCCGACTTTATCAC	AGAP003898_R	TAATACGACTCACTATAGGGTTTGCTCCAGCGTCTCTC
A12	AGAP003960	AGAP003960_F	TAATACGACTCACTATAGGGCCCACTGTTTCAGTAACG	AGAP003960_R	TAATACGACTCACTATAGGGCGCAGCGGGTAGTAGATAG
A13	AGAP004017	AGAP004017_F	TAATACGACTCACTATAGGGCGCATTCCTGCAGCATACTA	AGAP004017_R	TAATACGACTCACTATAGGGCCAGGTACCGTAAGGCGTAA
A14	AGAP004928	AGAP004928_F	TAATACGACTCACTATAGGGCCTCCGACAATAAGCGTCAT	AGAP004928_R	TAATACGACTCACTATAGGGCCGTCCCAAATATGCTTTA
A15	AGAP004993B	AGAP004993B_F	TAATACGACTCACTATAGGGCTTGGAGCTTGAAGGTGGAG	AGAP004993B_R	TAATACGACTCACTATAGGGTGAGCGTAGCGTTACGAATG
A16	AGAP004993C	AGAP004993C_F	TAATACGACTCACTATAGGGCAAGCTGGTAGTGGATA	AGAP004993C_R	TAATACGACTCACTATAGGGTGATCATCTCCAGCACGAAG
A17	AGAP005174	AGAP005174_F	TAATACGACTCACTATAGGGCTCAAACCAATCCTCGATCC	AGAP005174_R	TAATACGACTCACTATAGGGTGATTGTGCCTCGCTTGTAG
A18	AGAP005227	AGAP005227_F	TAATACGACTCACTATAGGGCCGAGTTCCAGATTGACGAT	AGAP005227_R	TAATACGACTCACTATAGGGCAGGCATTCCTTGGACATTT
A19	AGAP005549	AGAP005549_F	TAATACGACTCACTATAGGGATGACGAGTTCGATGCGTA	AGAP005549_R	TAATACGACTCACTATAGGGAGACAATCGGTTGGATCGAG
A20	AGAP005551	AGAP005551_F	TAATACGACTCACTATAGGGATTGCGCTGAACGAAGAGAT	AGAP005551_R	TAATACGACTCACTATAGGGTCGCACTCATCGTACCGTAG
A21	AGAP005992	AGAP005992_F	TAATACGACTCACTATAGGGTCGCTCATGGAGGAGTACCT	AGAP005992_R	TAATACGACTCACTATAGGGATGCACTGGTTCCTTTGT
A22	AGAP006769	AGAP006769_F	TAATACGACTCACTATAGGGTACACACTGCACGCACGTAA	AGAP006769_R	TAATACGACTCACTATAGGGCTCTTCTGGTGCGGAATCTC
A23	AGAP007499	AGAP007499_F	TAATACGACTCACTATAGGGACGTCGACAGTAGCGATGA	AGAP007499_R	TAATACGACTCACTATAGGGTTCAGCAGCGACCAGTACA
A24	AGAP007540	AGAP007540_F	TAATACGACTCACTATAGGGTACCATCAGCTGGCCATACA	AGAP007540_R	TAATACGACTCACTATAGGGACACATGAACGACAGCTTGC
A25	AGAP008086	AGAP008086_F	TAATACGACTCACTATAGGGCTGAACCGGTACCACTGGAT	AGAP008086_R	TAATACGACTCACTATAGGGTGTCGCTTGGAGAAGGCTAT
A26	AGAP008492	AGAP008492_F	TAATACGACTCACTATAGGGTTCTGCAGCAACATCTGACC	AGAP008492_R	TAATACGACTCACTATAGGGAAACGTTGCGATATCCTTGG
A27	AGAP008521	AGAP008521_F	TAATACGACTCACTATAGGGTCGATCTCACCGAAGGGTA	AGAP008521_R	TAATACGACTCACTATAGGGCCGTCAGCTCGGAGTTAAAA
A28	AGAP008908	AGAP008908_F	TAATACGACTCACTATAGGGTCATCAAGGACATCGAGCAG	AGAP008908_R	TAATACGACTCACTATAGGGCAGGTTGGTGTCCGAGTTTT
A29	AGAP008909	AGAP008909_F	TAATACGACTCACTATAGGGAGCATGGAGAAGCACGAAGT	AGAP008909_R	TAATACGACTCACTATAGGGAGCTGGTACTTCTCGGCAAA
A30	AGAP009119	AGAP009119_F	TAATACGACTCACTATAGGGCTGGAAATTAACCCCGACT	AGAP009119_R	TAATACGACTCACTATAGGGTCATCAGCACCGACATGATT
A31	AGAP009200	AGAP009200_F	TAATACGACTCACTATAGGGACTATCCGCACAACCAGGAC	AGAP009200_R	TAATACGACTCACTATAGGGAACCAGCCAGAAGGAGGTTT
A32	AGAP009201	AGAP009201_F	TAATACGACTCACTATAGGGATGCCGTTCATGTTCTGTGA	AGAP009201_R	TAATACGACTCACTATAGGGTCAATGATCGTCAGCCAGA
A33	AGAP009459	AGAP009459_F	TAATACGACTCACTATAGGGAGATCGTGACGCTGTGGTA	AGAP009459_R	TAATACGACTCACTATAGGGAAGGAATGGCAAAACACCTG

A34	AGAP009792	AGAP009792_F	TAATACGACTCACTATAGGGTACGTGACGGACGATGATG	AGAP009792_R	TAATACGACTCACTATAGGGTCGACGAGCTGAATGATCTG
A35	AGAP010580	AGAP010580_F	TAATACGACTCACTATAGGGCAGGGTGTTGTCCTCCTGTT	AGAP010580_R	TAATACGACTCACTATAGGGCTTCTTGGGCATCATCGTTT
A36	AGAP010587	AGAP010587_F	TAATACGACTCACTATAGGGCAATGGAATGAAGCAAAGCA	AGAP010587_R	TAATACGACTCACTATAGGGTAGGAGATGAGCGTGAGC
A37	AGAP011223	AGAP011223_F	TAATACGACTCACTATAGGGTGGCTAGTGATTCAGCATCG	AGAP011223_R	TAATACGACTCACTATAGGGTGCCAATCCCTGGTAAGAAG
A38	AGAP011984	AGAP011984_F	TAATACGACTCACTATAGGGCGGTGGTAAGCGATATGAT	AGAP011984_R	TAATACGACTCACTATAGGGCGGTACTGGAATGTGTCGTG
A39	AGAP012034	AGAP012034_F	TAATACGACTCACTATAGGGTCATGCCCGACTGAATGATA	AGAP012034_R	TAATACGACTCACTATAGGGTGTACTCTGACACCCGCATC
A40	AGAP012614	AGAP012614_F	TAATACGACTCACTATAGGGACCCCTCACGCTTTATGTTG	AGAP012614_R	TAATACGACTCACTATAGGGAGTTGCCAAATCCCACTCAC
A41	AGAP010658	AGAP010658_F	TAATACGACTCACTATAGGGCGAAGATGCACTCCCTTAGC	AGAP010658_R	TAATACGACTCACTATAGGGCGTGTACGTTGCATCAAAGG
A41-I	AGAP010658	AGAP010658_F1	TAATACGACTCACTATAGGGCGTTCTGCCACGGTACCTAT	AGAP010658_R1	TAATACGACTCACTATAGGGCATACCCTCGTTGAGGTTGG
LacZ		LacZ_T7_F	TAATACGACTCACTATAGGGAGAATCCGACGGGTTGTTACT	LacZ_T7_R	TAATACGACTCACTATAGGGCACCACGCTCATCGATAATTT
	AGAP010592	AgS7_qF	GTGCGCGAGTTGGAGAAGA	AgS7_qR	ATCGGTTTGGGCAGAATGC
	AGAP004928	A14_QF	GGAAGCATCGAAAAGAGTGC	A14_QR	CCGCAAAATTTACCCCTTTT
	AGAP005227	A18_QF	CCACAAGCACGAGATTGAAA	A18_QR	ACATTGCCCACACTGTCAAA
	AGAP009201	A32_QF	AGGTCGGACTCGAAGGCTA	A32_QR	TGGACAGGTTGGGCAGAT
	SNAP_*017730	A41_QF	TTAGCAATGGGCTTGGATTC	A41_QR	GGTTCAAGCGTTGCTTTCTC
	AGAP010658	A41-I_QF	GAAATTCATCCGCACCTGTT	A41-I_QR	CGTAGTTCGGGTCAAGTGCT

Additional Table S2. Columns report dsRNA name, AGAP identification code, primer forward and reverse names and primers sequences. In the upper part of the Table, primers used for dsRNA synthesis are listed, while in the bottom of the Table are reported details of primers used in real-time RTqPCR (q primers) experiments. *: ANOPHELES00000.
