# Supplementary material for: Novel factors of Anopheles gambiae haemocyte immune response to Plasmodium berghei infection
Source: Parasit Vectors. 2016 Feb 9;9:78. doi: 10.1186/s13071-016-1359-y (PMC4746906; doi:10.1186/s13071-016-1359-y)
Supplement: Additional file 4: Table S3. — Measurement of knock down efficiency. (PDF 53 kb) [file 13071_2016_1359_MOESM4_ESM.pdf]

Additional Table S3. Measurement of knock down efficiency.

ID	Publication	KD efficiency (REL, %)	KD phenotype *in vivo*
AGAP004928	Lombardo *et al*., 2013	33 ± 5	yes
AGAP005227	Lombardo *et al*., 2013	43 ± 18	no
AGAP009201	Lombardo *et al*., 2013	77 ± 14	yes
SNAP_*017730	This manuscript	23 ± 3	yes
AGAP010658	This manuscript	20 ± 1	no

ID: AGAP ID; * ANOPHELES00000.
Publication: source of the information [Lombardo F. *et al*. (2013), PLoS Pathog 2013, 9(1):e1003145].
KD efficiency (REL, %): effect of silencing on Relative Expression Level (REL) of the target gene in dsRNA treated mosquitoes compared to dsLacZ treated mosquitoes.
KD phenotype *in vivo*: occurrence of a modulation of *Plasmodium* development in KD mosquitoes.

Additional Table S3. Measurement of knock down efficiency. Knock down efficiencies were assessed for a group of genes selected from the haemocyte-specific dsRNA library used for *in vivo* screening (39 dsRNA). Percentage of expression of silenced gene is reported as Relative Expression Level of the target gene in dsRNA treated mosquitoes compared to dsLacZ treated mosquitoes.
